# Honey as a Wound Care Modality in Treating Deep Neck Space Abscesses: Protocol for a Randomized Controlled Trial

**DOI:** 10.2196/75475

**Published:** 2025-08-14

**Authors:** Dian Paramita Wulandari, Yanri Wijayanti Subronto, Agus Surono

**Affiliations:** 1 Department of Otorhinolaryngology Head and Neck Surgery Faculty of Medicine, Public Health, and Nursing Universitas Gadjah Mada Sleman Indonesia; 2 Department of Internal Medicine Faculty of Medicine, Public Health, and Nursing Universitas Gadjah Mada Sleman Indonesia

**Keywords:** natural dressing, cytokines, growth factor, Bates-Jensen Wound Assessment tool, clinical trial protocol, honey

## Abstract

**Background:**

Deep neck abscesses are a disease in the field of otorhinolaryngology-head and neck surgery that causes significant morbidity, death, and expenditures. Treatment length, whether inpatient or outpatient, is also prolonged. Deep neck abscesses are managed with incision and drainage, abscess exploration, systemic broad-spectrum antibiotic treatment, comorbidity control, and postoperative wound care through recovery. Standard dressings for wound treatment have proven time-consuming and expensive. Honey is one type of dressing that has long been used in wound treatment for a number of body areas and disorders.

**Objective:**

The purpose of this study is to investigate the use of honey as a potential substitute for standard dressings for deep neck abscesses.

**Methods:**

This is a single-blind randomized controlled trial. Randomization will be done through simple random sampling. The population and sample of the study include all patients with deep neck abscesses treated at Dr Sardjito General Hospital, which is equipped with board-certified otorhinolaryngologists. Patients with deep neck abscesses who were given ethical clearance and meet the inclusion criteria were recruited as study participants until the sample size was reached. There are 18 participants in each group. Participants in the intervention group received standard dressings in addition to honey dressings, whereas those in the control group received standard dressings alone. Proinflammatory cytokines and growth factors, wound size, Bates-Jensen Wound Assessment Tools scoring, and quantitative bacterial colony identification will be all evaluated and assessed. The gathered data will be documented and subjected to statistical analysis.

**Results:**

This manuscript presents a study protocol for a randomized controlled trial investigating honey as a wound care modality in patients with deep neck space abscesses. Ethical approval and partial funding for the study were obtained in May 2024. Recruitment and data collection commenced in June 2024 and were successfully completed as of June 2025.

**Conclusions:**

We hypothesize that honey may serve as a safe, effective, and affordable alternative wound dressing for the management of deep neck abscesses, with potential benefits in both clinical and health care system outcomes.

**Trial Registration:**

ClinicalTrials.gov NCT06562257; https://clinicaltrials.gov/study/NCT06562257.

**International Registered Report Identifier (IRRID):**

DERR1-10.2196/75475

## Introduction

### Background

The condition known as deep neck space infection (DNSI) emerges when an infection spreads into the deep neck spaces. If left undiagnosed and improperly treated, this condition may become life-threatening [1]. In Spain, there were 9-15 DNSI cases for every 100,000 people in 2012 [2]. DNSI has a 10% to 20% incidence rate and can lead to life-threatening complications if there is excessive accumulation of fluid in the deep neck space. It can also worsen into a deep neck abscess [3]. In Taiwan, there were 196 cases of DNSI between 1996 and 2002, with 33 cases per year, with a mortality rate of 7.6% [4]. DNSI cases increased from 1 case in 2006 to 15 cases in 2015 in Scotland [5]. Meanwhile, from 2004 to 2015, deep neck abscesses had a mortality rate of 1.4% and a complication rate of 22% in Finland [6]. The prevalence is higher in low- and middle-income countries, such as in Mexico, where DNSI has a prevalence of 42 cases over 5 years, with an average of 8.4 cases per year and a mortality rate of 8% [7], and in Ghana, where the prevalence is 135 cases over 7 years (2013-2020) with a mortality rate of 12.6% [8].

Between January 2017 and June 2020, the Otorhinolaryngology-Head and Neck Surgery (ORL-HNS) Department of Dr Sardjito Hospital in Indonesia had the highest number of DNSI cases (24) and the lowest number (8) per semester. With an average rate of mortality of 15% between 2018 and 2020, this number has been rising annually. For instance, in 2021, there were up to 41 patients with DNSI; in 2022, the total spiked to 63 patients; and in November 2023, there were 50 patients [9]. This mortality rate is comparatively greater than that of Bandung and other countries [9]. The average mortality rate was 12.4% from 2015 to 2019 [9]. According to all of the prior studies, men are more frequently affected than women, and while the age range varies from early childhood to adults, the majority of patients are adults.

A deep neck space abscess (DNSA) is an abscess that establishes within the space between the deep neck fasciae. The rapid progression of tonsillar and pharyngeal infections brought on 70% of DNSIs prior to the invention of antibiotics, which frequently led to parapharyngeal abscesses. Infections in adults generally are of dental or odontogenic origin; however, tonsillar infections remain the most common cause in young adults. Although the prevalence and mortality of DNSIs have significantly decreased due to antibiotics, hospital cases of these infections are still frequent [10-12].

Incisional drainage, abscess exploration, systemic broad-spectrum antibiotic therapy, comorbid factor control, and postoperative wound care until the wound closes are the methods used to treat DNSAs. Because a DNSA is situated between the fasciae, the wound has a deeper shape and location and heals at a different rate than other wounds. After DNSA investigation, the wound typically takes a long time to heal due to the complicated nature of treatment methods; the longest healing period can be up to 4 months, with an average of 14 days before the wound closes [13]. In addition to supplementary costs like medical procedures, surgery, consultation, and interdepartmental management, patients with DNSAs endure a significant health cost burden as a result of this prolonged wound healing.

A wound is defined as any discontinuity, disturbance, or disruption in the anatomical structure of the skin or connective tissue that results in functional impairment [14]. The wound healing process is divided into several stages, including homeostasis, inflammation, proliferation, and remodeling. These stages are modified by diverse cellular contacts and controlled by the secretion of local chemical signals [14].

The recovery process of a wound determines whether it is acute or chronic. The Centers for Disease Control and Prevention divides wounds into 4 classifications: clean, clean contaminated, contaminated, and dirty or infected. Postdrainage and abscess exploration wounds are dirty or infected open deep wounds that will heal through secondary wound healing.

In acute conditions, most surgeons continue to use sterile gauze with saline as the wound dressing of choice [15]. The wound irrigation solutions used include normal saline, tap water, povidone-iodine, Ringer lactate solution, hypochlorous acid, polyhexamethylene biguanide (PHMB), sodium hypochlorite, and electrolyzed strong water acid. Although these solutions can be used as wound irrigation agents, only some of them have antibacterial activity. One of the bactericidal wound irrigation agents is PHMB, an antimicrobial polymer that is effective against intracellular and biofilm forms of *Staphylococcus aureus* [16]. PHMB is quite effective in the treatment of wounds in deep neck abscesses. However, the unit cost is relatively high and is frequently not covered by government insurance.

Wound dressings with honey have been practically used since ancient Egyptian times to treat abscesses, diabetes mellitus, eye and ear infections, gastrointestinal diseases, oral ulcers, gynecological diseases, nail infections, airway infections, and wounds after circumcision [17]. Honey has also been used since the wartime era of the Prophet Muhammad to treat wounds. It was later shown that honey can kill bacteria in wounds and can be used as a medicine. This method is still used today in several Islamic countries to heal wounds and has been used by Russian soldiers since World War I to prevent wound infection and accelerate wound healing [18].

Honey has a high sugar content and high acidity, which can reduce bacterial growth. It also contains hydrogen peroxide, which is capable of eradicating pathogenic microbes, as well as antibacterial chemical compounds. Honey is more readily obtainable and less expensive than other dressing materials.

### Goal of the Study

Honey contains antibacterial components that can help treat wound infections, relieve pain, and promote circulation, which all contribute to the healing process. Studies on the use of honey in wound care have primarily been conducted on superficial wounds as well as diabetic ulcers and burns. Previous clinical trials on the use of honey in wound care looked at applying honey to wounds after excision of pilonidal cysts [19]. Research by Rai et al [20] in 2023, which included a randomized controlled trial, compared the use of honey and Edinburgh University’s solution of lime in necrotizing fasciitis wounds. Research by Dubhashi and Sindwani [21] in 2015 looked at the comparative use of honey and phenytoin in chronic wounds. In 2017, Esser [22] examined the use of leptospermum honey on wound healing on the skin of premature newborns admitted to the neonatal intensive care unit. In 2007, Güneş and Eşer [23] compared honey dressings and ethoxy-diamino acridine plus nitrofurazone dressings in patients with decubitus ulcers and assessed healing with the pressure ulcer scale for healing. Wahdini et al [24] reported the use of Madu Nusantara, an Indonesian honey, as a wound dressing for extensive diabetic ulcers in 1 patient with a history of uncontrolled type 2 diabetes mellitus, with good results. Saputri et al [25] from Dr Hassan Sadikin Hospital also reported a case of using Madu Nusantara as a wound dressing in a patient with a submandibular abscess with necrotizing fasciitis. Notably, clinical trials assessing the use of honey on patients with deep neck abscesses have never been conducted before. The mechanistic action of honey in wound healing has been widely hypothesized; however, no one has evaluated its effect clinically, systemically, or on the proinflammatory cytokines and growth factors that play a role in each phase of wound healing.

Thus, this study will conduct a clinical trial of honey as one of the modalities in the treatment of deep neck abscess wounds. Honey is expected to become an alternative dressing material in the treatment of deep neck abscess wounds due to its effectiveness, safety, availability, and affordability compared to standard dressings. Furthermore, honey is readily obtainable at an affordable price; therefore, if the results of wound care using honey are positive, this work will significantly reduce the burden of care on patients, doctors, and hospitals.

## Methods

### Study Design

This study is an experimental, quantitative study to determine the effectiveness of honey in treating deep neck abscess wounds. The research design used was a single-blind randomized controlled trial, and the intervention was given randomly to the research participants. This protocol follows the SPIRIT (Standard Protocol Items: Recommendations for Interventional Trials) 2013 guidelines [26].

### Recruitment of Participants

This study investigated wound care strategies for patients with deep neck abscesses. The research design used a single-blind randomized controlled trial comparing honey dressings with standard dressings (Prontosan irrigation solution). The study population included patients treated at Dr Sardjito Hospital, Yogyakarta. Patient recruitment was conducted from the date of ethical clearance issuance until the required sample size was achieved. Before enrolment, all patients provided written informed consent after being fully informed of the study objectives, procedures, potential risks, and benefits. The study protocol was approved by the Medical and Health Research Ethics Committee, Faculty of Medicine, Public Health and Nursing, Universitas Gadjah Mada, Dr Sardjito General Hospital (KE/FK/0668/EC/2024), ensuring compliance with national and international ethical standards. The inclusion criteria included patients aged older than 18 years with or without comorbidities, except for malignant diseases. The exclusion criteria excluded patients who refused the intervention, underwent vacuum-assisted closure, or had incomplete medical records. Severe allergic reactions to dressings were considered withdrawal criteria. Our findings contribute to the optimization of wound care practices for deep neck abscesses, aiming for improved outcomes and cost-effectiveness. Our investigation was held at the ORL-HNS Department in Dr Sardjito General Hospital.

### Sample Size

The sample size in this study was calculated based on the formula for unpaired categorical-numerical comparative research involving a single numerical measurement between 2 groups [27]. The parameters used included a significance level (α) of .05 and a power (1−β) of 90%, corresponding to Zα=1.64 and Zβ=1.28, respectively. According to previous studies, the mean (SD) wound closure in the control group (standard dressing) was 0.266 (SD 1.183) cm. Meanwhile, the expected mean wound closure in the intervention group (honey dressing), based on prior research using honey in wound management, is 1.433 cm. Using these values, the estimated minimum sample size required was approximately 18 participants per group. The full calculation procedure is provided in Multimedia Appendix 1 [21].

### Randomization

In the study we conducted, participants were assigned to the control and intervention groups using a comprehensive randomization technique. The distribution was carried out by concealing opaque envelopes with numbered slips ranging from 1 to 36. These slips were arranged in a box, and the sequence was chosen using the Microsoft Excel random generator. Importantly, the researchers remained blind to the randomization findings throughout the process.

The participants were then assigned to either the control group (standard dressing) or the intervention group (standard dressing plus Indonesian Nusantara honey). The resident doctor was responsible for administering the dressing and obtaining detailed randomization instructions.

### Blinding

This study was designed as a single-blind clinical trial. The principal investigator and the assistant who measured wound indicators remained blinded throughout the treatment period, and treatment allocation was disclosed only after the final database lock. Patients were not blinded, as full blinding was not feasible due to the intrinsic characteristics of honey. Its distinct color may stain the dressing material, and its recognizable odor may reveal the intervention. These factors could have led patients to infer their group allocation, making participant blinding impractical.

### Honey Sample

Honey has an extended record of enabling wound healing and offers a more affordable therapeutic option. This study used raw and unsterilized local honey known as Nusantara honey. Nusantara honey contains biological components and beneficial microorganisms that promote wound healing. As a result, the honey used in this study was not sterilized, as sterilization can diminish its benefits. The honey used in the study has already passed Indonesia’s food and drug administration test (known as BPOM) with the number MD 011167000300234. The same batch of honey is used in the study, ensuring the quality and safety of the product remain the same. It was confirmed to be safe because it passed the laboratory test at the Health Laboratory Calibration and Testing Center, Regional Health Office of Yogyakarta. The test results showed negative findings for fungal and bacterial cultures. The certificate, issued under number 009008/LHU/BLKK-Y/05/2024, confirmed that the honey sample met the safety standards for clinical application. Although sterilization was not performed to preserve its bioactive components, the honey was certified safe and free from harmful contaminants. This was validated by the extraction of samples following wound healing, where laboratory studies revealed the absence of toxins, fungus, or potentially hazardous bacteria.

In several previous studies, honey was used clinically, and the same brand has been applied in Indonesia. In studies in other countries, natural honey has been used without sterilization and has been shown to produce better results [23,28-35].

### Intervention Procedure

Both the control and intervention groups received wound care following the standard operating procedure established by the Laryngopharyngology Division, ORL-HNS Department, Dr Sardjito General Hospital. This protocol includes wound debridement, saline irrigation, application of gauze rolls, and administration of Prontosan. Necrotic tissue was removed, and the wound was disinfected using sodium chloride before a gauze roll soaked with antibiotic ointment was inserted to serve as drainage. Dressing frequency was determined based on the volume of pus produced—daily dressing was performed until pus production decreased to less than 5 cc, at which point dressings were changed every 3 days, eventually decreasing to once a week when no pus was observed.

In the intervention group, the standard protocol was followed with an additional intervention using Nusantara honey. A 120-cm gauze roll was immersed in 12 cc of Nusantara honey for approximately 1 minute, allowing full absorption before being applied to the wound and covered with sterile gauze. Prontosan gel was used when pus was no longer present to stimulate granulation tissue formation until the wound base reached the surface. Wounds were generally left to heal by secondary intention, unless primary closure or reconstruction was necessary due to extensive tissue loss.

Monitoring of adverse events was conducted directly by the principal investigator and otorhinolaryngology residents during each wound dressing session. If the honey dressing failed to achieve adequate wound healing outcomes, a swab was taken from the wound base for repeat sensitivity culture. The wound care protocol was then reverted to the standard dressing procedure used at Dr Sardjito General Hospital, followed by further management based on the patient’s condition, including the treatment of any comorbidities that might hinder the healing process. The antibiotic protocol applied to both the control and intervention groups included ceftriaxone and metronidazole upon initial presentation, with adjustments made according to the culture results when available. In cases of infection escalation, antibiotics were modified following the hospital’s infection control guidelines. The average wound healing time was 7 days, although some cases required up to a month or additional surgical interventions, such as grafting.

### Measurements

Table 1 outlines the schedule for data collection. All participants who were intervened on had data collected on days 1, 7, and 14. The data comprised measurements of bacterial colony counts, wound size, Bates-Jensen Wound Assessment Tools (BWATs), proinflammatory cytokines, and growth factors. The data are intended to reflect the effects of interventions performed on wound healing in each stage.

**Table 1:** Schedule for data collection.

Activity		Timeline (days)
Filling out the case report form		1, 7, and 14
Wound measurement		1, 7, and 14
Measuring BWATs^a^ (size, depth, edges, undermining, necrotic type, necrotic amount, exudate type, exudate amount, skin color, edema, induration, granulation, and epithelialization)		1, 7, and 14
Taking a wound bed swab		1 and 14
Taking wound tissue (biopsy)		1 and 14
Vital sign examination (blood pressure, respiratory rate, heart rate, temperature, SPO2^b^, and VAS^c^)		1-14 (daily)
**Supporting examinations**		
	Complete blood count^d,e^	1-14 (daily)
	Computed tomography scan^e^	1 and 14
	Chest X-ray^e^	1 and 14
	Blood gas analysis^e,f^	1, 4, 7, 10, and 14
Monitoring complications		1-14 (daily)
Pus culture aspiration (bacterial, sensitive to antibiotic, intermediate to antibiotic, or resistant to antibiotic)		1 and 14
Wound bed culture (bacterial, sensitive to antibiotic, intermediate to antibiotic, or resistant to antibiotic)		1 and 14
Measurement of growth factors and cytokines (IL-1, TNFα, and VEGF)		1 and 14

^a^BWAT: Bates-Jensen Wound Assessment Tool.

^b^SPO2: saturated oxygen.

^c^VAS: visual analog scale.

^d^Complete blood count includes hemoglobin, red blood cells, white blood cells, and platelets. Other laboratory tests include blood ABO group and rhesus type, prothrombin time, activated partial thromboplastin time, international normalized ratio, total bilirubin, direct bilirubin, indirect bilirubin, serum glutamic oxaloacetic transaminase, serum glutamic pyruvic transaminase, albumin, sodium, potassium, chloride, blood urea nitrogen, urea, creatinine, uric acid, random blood glucose, fasting blood glucose, 2-hour postprandial glucose, hemoglobin A1C, and hepatitis B surface antigen.

^e^As long as the patient was still admitted in the ward.

^f^Arterial blood gas parameters include pH, partial pressure of carbon dioxide, bicarbonate, partial pressure of oxygen, and base excess.

Each parameter was measured following standardized procedures, and designated personnel were assigned to each task to minimize measurement bias and ensure internal validity.

On day 1, the following assessments were performed:

- Wound size (length, width, and depth): Wound size was measured by a trained research assistant. The measurement was conducted with a specific ruler and probe. The width and length recorded were the largest calculated amounts, whereas the depth of the wound was measured using a specific probe inserted perpendicularly to the meeting point of the length and width until it reached the base of the wound. To enhance measurement precision and reproducibility, the ImitoMeasure wound documentation app was also used [36]. This mobile app enables photographic documentation with calibrated scale indicators, providing consistent digital measurements of wound area and depth. This measurement was conducted by the research assistant 3 times, and the results were averaged to obtain a more accurate wound size estimate.
- BWAT: The raw wound data and clinical wound appearance were first recorded by an assistant. The final scoring and interpretation based on these observations were conducted independently by the principal investigator, who was blinded to treatment allocation during the evaluation process.
- Wound swabs for bacterial culture: Infection was considered to have improved as evidenced by bacterial culture findings and the number of bacterial colonies that proliferated. Samples were collected by the clinical team under sterile conditions and submitted to a certified microbiology laboratory for quantitative colony count analysis. Samples were taken from the wound base using sterile cotton, placed in Amies transport medium, and delivered to a microbiological laboratory.
- Tissue biopsy for cytokine (IL-1 and TNF-α) and growth factor (VEGF) analysis: Tissue biopsy was performed by an otorhinolaryngology resident using standard sterile technique. The biopsy samples were then processed by trained laboratory personnel for immunohistochemical examination.

On day 7, wound size measurements and BWAT scoring were repeated using the same tools and by the same assessors as on day 1 to ensure consistency across time points.

**Figure 1:**
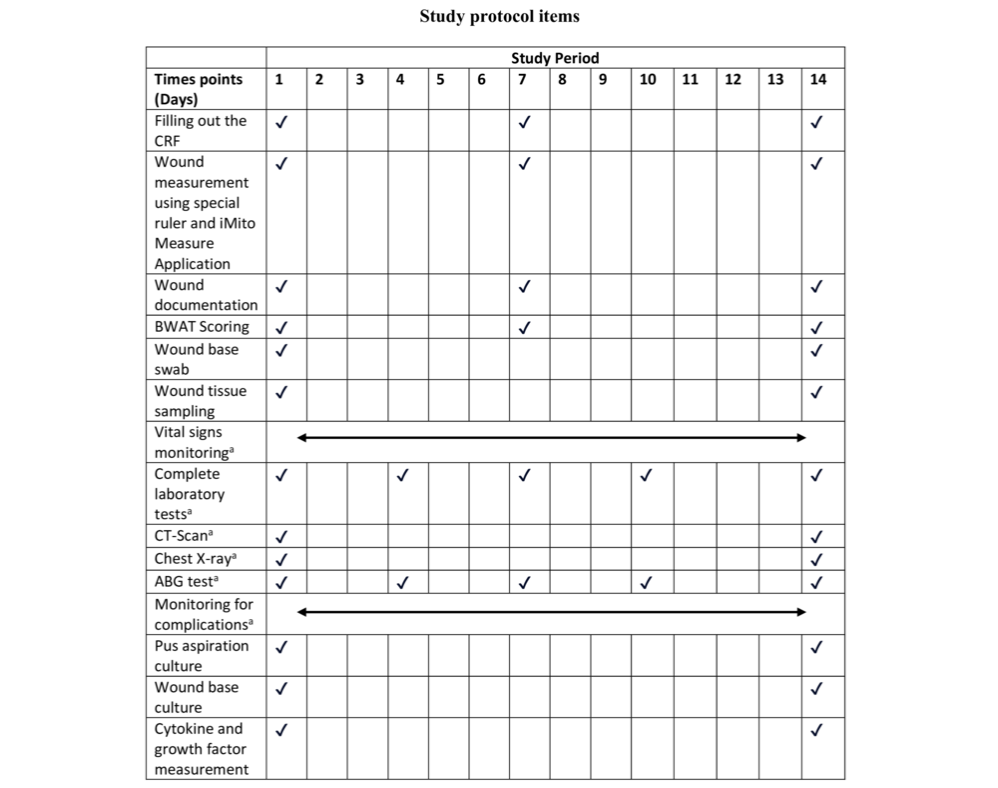
Study protocol items and schedule of assessments during the 14-day observation period. The tests will be conducted as long as the patient is hospitalized.

On day 14, all baseline measurements were repeated, including wound size, BWAT scoring, wound swab cultures, and tissue biopsies for cytokine and growth factor quantification. The same assigned personnel performed all assessments to reduce interobserver variability and enhance the reliability of longitudinal comparisons. The schedule of interventions and sample measurements is illustrated in Figure 1.

### Outcomes

In this study, a research assistant collected samples after completing wound dressing and storing the dressing materials. Measurements were taken on the first, seventh, and fourteenth days for BWAT assessments, as well as the first and fourteenth days for bacterial colony identification from wound bed swabs and cytokine and growth factor expression analysis from tissue samples. The resident doctor was accountable for the dressing and performed pus aspiration and tissue sampling. All information was meticulously documented on an authorized form. Wound measures included length, width, and depth, with data based on the largest width and length. Wound depth was measured using a probe perpendicular to the wound’s length and width. BWATs were evaluated by an uninformed research assistant. Bacterial culture findings and the number of bacterial colonies were used as evidence of infection improvement. Swabs from the wound bed were delivered to the microbiology lab. Tissue samples were sent to the Anatomical Pathology lab for immunopathological examination of wound healing, including quantification of IL-1, TNF-α, and VEGF expression. Tissue samples were collected using nasopharyngeal biopsy forceps, and the preserved tissue was sent out for analysis. Additionally, laboratory examinations, radiographs, and patient comorbid factors were documented for analysis in the case report form.

### Statistical Analyses

**Figure 2:**
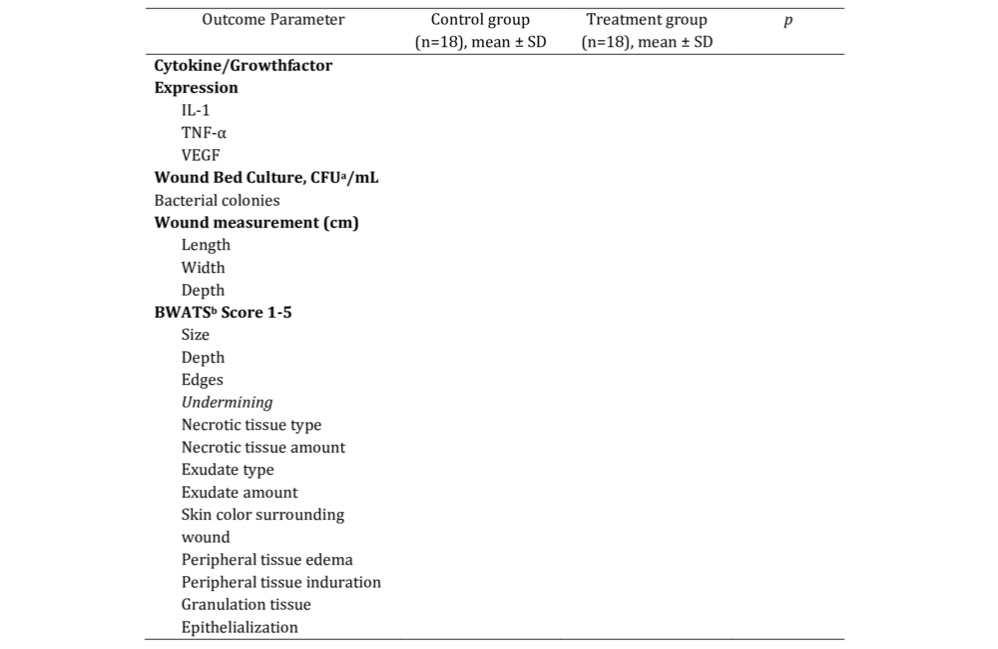
Sample table of outcome parameters to be collected on days 1, 7, and 14. a CFU: Colony forming unit b BWATs: Bates Jensen Wound Assessment Tool.

The Shapiro-Wilk test will be used to assess the normality of continuous data because the sample size is less than 50. For comparison of wound healing outcomes between the honey dressing and standard dressing groups at each time point (days 1, 7, and 14), either an independent *t* test or Mann-Whitney U test will be used, depending on data distribution. To analyze the overall effect of treatment over time and assess the interaction between treatment group and time, a generalized estimating equation analysis will be performed. A *P* value of <.05 will be considered statistically significant. All data will be analyzed using SPSS software version 25.0 (IBM Corporation) and presented in tables summarizing the changes on days 1, 7, and 14 (Figure 2).

## Results

**Figure 3:**
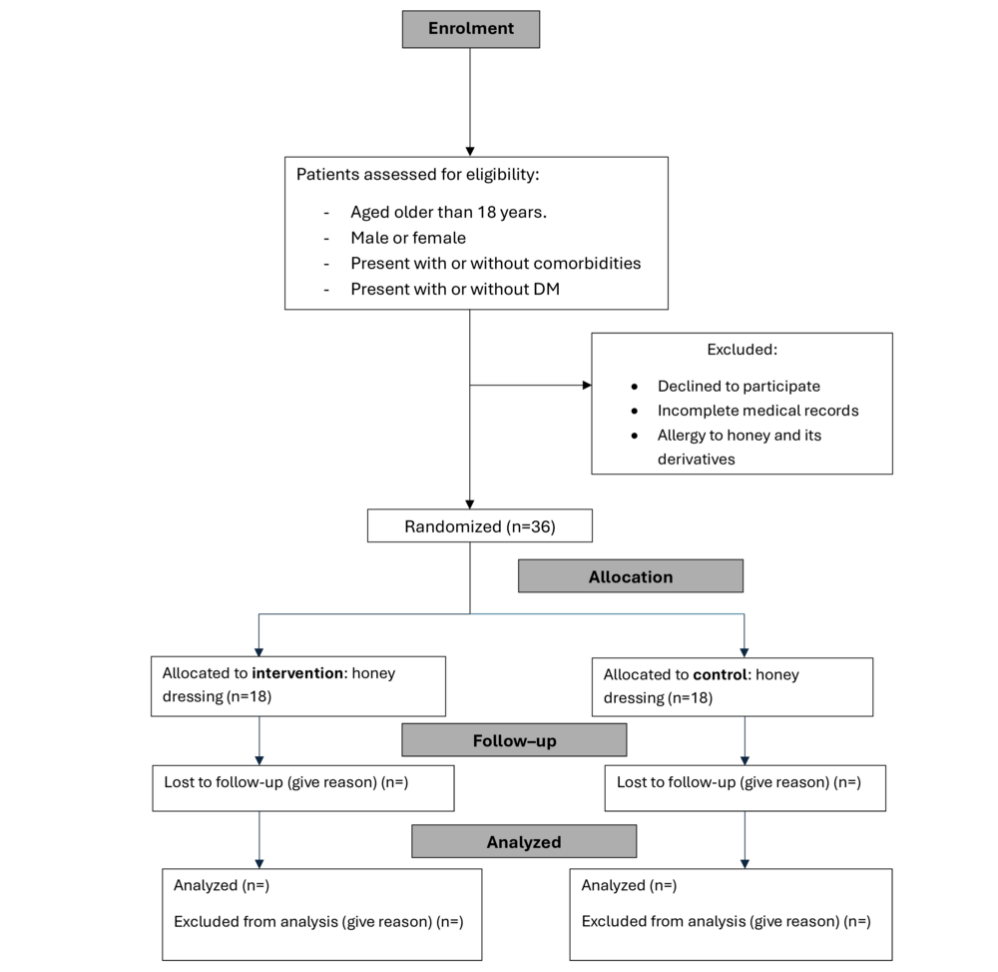
CONSORT (Consolidated Standards of Reporting Trials) flowchart of study participants.

This manuscript presents a study protocol for a randomized controlled trial investigating honey as a wound care modality in patients with DNSAs. Ethical approval and partial funding for the study were obtained in May 2024. Recruitment and data collection commenced in June 2024 and was completed in May 2025. As of manuscript submission, the full sample of 36 participants has been successfully enrolled (Figure 3). Data analysis is currently underway, and the study results are expected to be submitted for publication in early 2026.

## Discussion

### Anticipated Findings

This study is designed to evaluate whether honey-based dressings can enhance wound healing in patients with deep neck abscesses compared to standard dressings. The anticipated findings are that honey dressings, due to their antibacterial, anti-inflammatory, and immunomodulatory properties, will result in faster and more effective healing outcomes. These outcomes will be assessed clinically, bacteriologically, systemically, and through biomarker analysis of IL-1, TNF-α, and VEGF expression.

Honey has long been used in traditional medicine, and modern research has supported its effectiveness in wound care. Previous studies have demonstrated honey’s ability to promote healing in burns, surgical wounds, and chronic ulcers. Its unique properties—such as high osmolarity, low pH, hydrogen peroxide production, and rich content of antioxidants and bioactive compounds—contribute to its antimicrobial and healing potential [17,18,37]. Particularly, Manuka honey has shown strong efficacy against drug-resistant bacteria, including methicillin-resistant *Staphylococcus aureus* and vancomycin-resistant *Enterococcus*, and has been successfully used in chronic wound treatment [17,38]. In vitro studies have also indicated that honey stimulates the release of inflammatory cytokines such as IL-1, TNF-α, and IL-6, which play essential roles in wound healing [37]. Despite these promising results, there remains a lack of clinical studies specifically examining the use of honey for deep neck infections, which are serious, rapidly progressing conditions with potential airway and systemic complications [2,3,6].

The strength of this study lies in its comprehensive design, integrating clinical, microbiological, and molecular assessments within a randomized controlled trial framework. This design allows for a multifaceted evaluation of honey’s effects on wound healing while minimizing potential bias. Nonetheless, certain limitations must be acknowledged. The use of raw honey, although supported by prior literature, poses concerns regarding microbial contamination. Bacterial spores, such as *Clostridium* and *Bacillus*, may be present in raw honey, although gamma irradiation is an effective method of sterilization that preserves its antibacterial properties [17]. Moreover, variation in honey composition depending on its botanical source may influence treatment outcomes, and the accessibility of standardized, medical-grade honey remains limited in some settings [18].

Should the study yield positive results, future research should explore the standardization of honey-based dressings and assess their scalability and cost-effectiveness in clinical practice. Comparative studies between different types of honey may also help to identify the most effective formulations for wound care. Further investigation into long-term outcomes, including the quality of scar formation and infection recurrence rates, would provide valuable insight into the broader benefits of honey in wound management.

The findings from this study will be disseminated through publications in peer-reviewed journals and presentations at scientific conferences focusing on otorhinolaryngology, wound care, and integrative medicine. In addition, efforts will be made to share the results with local hospital networks and health care providers to inform evidence-based clinical practices. Educational materials for clinicians and patients may also be developed to promote the safe and effective use of honey in wound management.

### Conclusion

In conclusion, this study proposes honey as a potential alternative wound dressing for the management of deep neck abscesses. Based on its established antimicrobial, anti-inflammatory, and wound-healing properties, honey may offer a safe, accessible, and cost-effective solution compared to conventional dressings. If proven effective through this trial, honey-based dressings could contribute to improved clinical outcomes and reduced health care burdens, particularly in low-resource settings.

## Appendix

Multimedia Appendix 1 Sample size calculation for determining the minimum number of participants per group.

## Abbreviations

CONSORT: Consolidated Standards of Reporting Trials
DNSA: deep neck space abscess
DNSI: deep neck space infection
ORL-HNS: Otorhinolaryngology-Head and Neck Surgery
PHMB: polyhexamethylene biguanide
SPIRIT: Standard Protocol Items: Recommendations for Interventional Trials
