# Supplementary material for: Honey as a Wound Care Modality in Treating Deep Neck Space Abscesses: Protocol for a Randomized Controlled Trial
Source: JMIR Res Protoc. 2025 Aug 14;14:e75475. doi: 10.2196/75475 (PMC12395109; doi:10.2196/75475)
Supplement: Multimedia Appendix 1 [file resprot_v14i1e75475_app1.pdf]

Sample size

The minimum sample size in this study follows the minimum sample size formula for unpaired categorical-numerical comparative research of two groups of one measurement.

n = minimum sample size.

Zα = standard normal distribution value (Z table) at a certain α, for α = 0.05, then Z = 1.64.

Zβ = standard normal distribution value (Z table) at a certain β, for β = 0.10, then Z = 1.28.

S = standard deviation between the two groups

x1 - x2 = estimated difference in mean values in population 1 with population 2.

When S = mean standard deviation = 1.183 (Preliminary study)

X2 = mean wound closure in the control group = 0.266 cm (this is the deep neck abscess wound healing data that has been available in our hospital)

X1 = mean wound closure in the honey group = 1.433 cm (this is data on wound healing in other types of wounds with honey in a previous study).

Then the sample size is:

$$n1=n2=\left|2\left[\frac{(Z\alpha+Z\beta)s}{x1-x2}\right]^2\right.$$

$$=\left|2\left[\frac{(1{,}64+1{,}28)1.183}{1.433-0.266}\right]^2\right.$$

$$=\left|2\left[\frac{3.4456}{1.166}\right]^2\right.$$

= 2x8.73 =17,4

∼ 18 patients per group
